# Trial to Encourage Adoption and Maintenance of a MEditerranean Diet (TEAM-MED): a randomised pilot trial of a peer support intervention for dietary behaviour change in adults from a Northern European population at high CVD risk

**DOI:** 10.1017/S0007114521003986

**Published:** 2022-10-14

**Authors:** Claire T. McEvoy, Sarah Moore, Christina Erwin, Meropi Kontogianni, Sara Megan Wallace, Katherine M. Appleton, Margaret Cupples, Steven Hunter, Frank Kee, David R. McCance, Christopher C. Patterson, Ian S. Young, Michelle C. McKinley, Jayne V. Woodside

**Affiliations:** 1 Centre for Public Health, Queen’s University Belfast, Grosvenor Road, Belfast BT12 6BJ, UK; 2 Department of Nutrition and Dietetics, Harokopio University, Greece; 3 Department of Psychology, Faculty of Science and Technology, Bournemouth University, Fern Barrow, Talbot Campus, Bournemouth BH12 5BB, UK; 4 Regional Centre for Endocrinology and Diabetes, Belfast Health and Social Care Trust, Grosvenor Road, Belfast BT12 6BA, UK

**Keywords:** Mediterranean diet, Dietary behaviour change, Peer support, Pilot trial

## Abstract

Adhering to a Mediterranean diet (MD) is associated with reduced CVD risk. This study aimed to explore methods of increasing MD adoption in a non-Mediterranean population at high risk of CVD, including assessing the feasibility of a developed peer support intervention. The Trial to Encourage Adoption and Maintenance of a MEditerranean Diet was a 12-month pilot parallel group RCT involving individuals aged ≥ 40 year, with low MD adherence, who were overweight, and had an estimated CVD risk ≥ 20 % over ten years. It explored three interventions, a peer support group, a dietician-led support group and a minimal support group to encourage dietary behaviour change and monitored variability in Mediterranean Diet Score (MDS) over time and between the intervention groups, alongside measurement of markers of nutritional status and cardiovascular risk. 118 individuals were assessed for eligibility, and 75 (64 %) were eligible. After 12 months, there was a retention rate of 69 % (peer support group 59 %; DSG 88 %; MSG 63 %). For all participants, increases in MDS were observed over 12 months (*P* < 0·001), both in original MDS data and when imputed data were used. Improvements in BMI, HbA1c levels, systolic and diastolic blood pressure in the population as a whole. This pilot study has demonstrated that a non-Mediterranean adult population at high CVD risk can make dietary behaviour change over a 12-month period towards an MD. The study also highlights the feasibility of a peer support intervention to encourage MD behaviour change amongst this population group and will inform a definitive trial.

The Mediterranean diet (MD) is a diet consumed by those living in Mediterranean regions, characterised by a high consumption of fruits, vegetables, wholegrains, legumes, nuts and seeds, moderate amounts of dairy products, moderate amounts of red wine, low to moderate amounts of fish and poultry, and low amounts of red meat with olive-oil as the principal source of fat^(1)^. Increased adherence to MD has been associated with longevity^(2)^, a lower prevalence of several chronic diseases, including CVD and cancer, and better management of type 2 diabetes mellitus (T2DM)^(3–5)^. Evidence from systematic reviews, meta analyses and randomised controlled trials (RCT) suggests a protective effect of MD adherence on incidence of CVD or CVD-related mortality and T2DM^(6–9)^. However, a recent Cochrane review found that, although there have been a large number of studies investigating MD, the effects of MD adherence on CVD risk factors and occurrence in those with and without existing CVD is still unclear^(10)^ but should be elucidated by the results of currently ongoing studies.

Transferability and adoption of an MD beyond the Mediterranean region also remains a challenge. Specifically, accessibility of MD foods, such as extra-virgin olive-oil, and barriers to adoption due to culture, religion, traditional cooking practices and economic burden, need to be addressed in interventions aiming to increase MD adherence in non-Mediterranean populations^(11,12)^.

From the previously conducted clinical trials, successful dietary change towards an MD is usually possible with resource intensive interventions, mainly delivered by health professionals and usually in parallel with food provision^(8,13,14)^. However, to scale-up these interventions in larger samples or to introduce them at a population level, there is a need to develop and test less intensive interventions not requiring expert delivery. Previous research, primarily conducted within the diabetes field, has found that involving lay peers in the delivery of dietary behaviour change programmes could be an alternative and potentially low-cost way of promoting dietary change which is culturally acceptable^(15–17)^.

The Trial to Encourage Adoption and Maintenance of a MEditerranean Diet study (TEAM-MED) was a pilot trial implemented in Northern Ireland, aiming to explore methods of increasing MD adoption in a non-Mediterranean population at high risk of CVD. This 12-month pilot study aimed to explore the feasibility of a peer support intervention *v* a previously tested dietitian-led intervention^(8)^ to encourage MD behaviour change and to test recruitment strategies, retention and attrition, to inform the design of a definitive trial in which the clinical and cost-effectiveness of the peer support intervention will be tested. The development of the peer support intervention, which included consideration of previously identified barriers to adoption^(18)^, has already been published^(19)^.

The objectives of the TEAM-MED pilot trial that are addressed in the current paper, and as detailed in the published protocol^(20)^, were to

1. Test recruitment strategies and estimate retention/attrition rates
2. Estimate and compare the variability of Mediterranean Diet Score (MDS) over the 12-month study between the peer support intervention and other intervention groups
3. Estimate and compare the variability of biochemical markers of nutritional status and health markers over the course of the intervention, as for MDS, between the peer support intervention and other intervention groups
4. Estimate the sample size for a large-scale trial

Furthermore, in the current paper, the association between change in MD adherence and change in biochemical markers of nutritional status and health in the population as a whole will be presented in order to explore whether increased adherence to an MD is associated with CVD and T2DM risk factors in this population.

## Methods

The study protocol and detailed methodology have been published previously^(20)^. TEAM-MED was a 12-month pilot parallel group RCT designed to evaluate the feasibility of a community-based peer support intervention to encourage adoption and maintenance of an MD, compared with a dietitian-led intervention^(8,21)^ and a minimal support intervention (which served as a control group), in adults at high risk of developing CVD. Ethical approval was received from the Office for Research Ethics Committees Northern Ireland (HSC RECA; ref 13/NI/0152). The study protocol was registered on ControlledTrials.com (ID no. ISRCTN68779848).

### Recruitment and screening procedure

A variety of strategies was used to recruit participants. An interview with a member of the research team was conducted on several radio stations, and the majority of participants were recruited following this publicity. Furthermore, presentations were given to the public about MD and heart health; a poster advertising the study details was placed in various locations including hospitals, dental surgeries, pharmacies, healthy living centres and shopping centres in the greater Belfast area and an intranet advertisement was devised and circulated to employees of Queen’s University Belfast and local Health and Social Care Trusts, as well as being placed on websites such as Volunteer Now and Gumtree. Study details were also advertised in several church bulletins across Northern Ireland and on a local shopping centre website. In addition, clinicians working at a weekly hyperlipidaemia outpatient clinic at the Royal Victoria Hospital, Belfast, referred potentially eligible individuals to the researchers. A letter of invitation to take part in the study was also sent to interested individuals who participated in study developmental work, which has been published separately^(20)^. A number of GP surgeries agreed to participate via the Northern Ireland Clinical Research Network, through screening patient data and sending a letter of invitation to individuals meeting the inclusion criteria.

Contact details of the research team were provided with all advertisement material, and individuals who were interested in participating in the study contacted the researchers, who explained the details of the study and answered any questions. Individuals were then initially screened by telephone to determine if they were likely to be suitable for inclusion in the study. Individuals who were deemed likely to be suitable were provided with a participant information sheet detailing the purpose and design of the study, details of participant involvement and any potential risks of participation. A period of at least 72 h was then given before the individual was re-contacted by one of the researchers to answer any further questions and determine if they wished to participate in the study. Interested individuals were then invited to attend a screening visit to formally assess their suitability.

Screening visits took place in the Centre for Public Health. Initially, the researcher explained the study procedure in detail and provided an opportunity for the individual to ask questions. Informed written consent for study procedures and collection, handling and storage of biological samples was obtained from all study participants. A questionnaire to record demographics, health and medical history and stage of dietary change towards an MD^(22)^ was used together with CVD risk prediction charts^(23)^ and an assessment of participant’s baseline MD adherence using a modified version of an existing fourteen-item questionnaire from the PREDIMED study^(8)^ to assess study eligibility.

### Inclusion criteria

Participants were included if they were aged 40 y or over, had a low adherence to MD (MDS of ≤ 3)^(13)^, had BMI > 27 and < 45 kg/m^2^ and had a combination of risk factors, according to the Joint British Societies CVD risk prediction charts, which would place them at an estimated multifactorial CVD risk ≥ 20 % over ten years (according to JBS score)^(23)^. Where there was no access to the clinical history at screening necessary to calculate JBS score, the study team determined > 20 % risk using (i) calculated JBS score > 20 % where all information was available or (ii) JBS score > 10 % plus 2 or more additional CVD risk factors (clinical obesity, abdominal obesity and family history of CVD).

### Exclusion criteria

Exclusion criteria were any established CVD or diabetes mellitus; surgery within the previous 3 months; pregnant or lactating; excessive alcohol consumption; psychiatric problems; medical conditions, allergies or dietary restrictions that would substantially limit ability to complete the study requirements; consumption of high-dose nutritional supplements; low predicted likelihood to change dietary habits (assessed using the stage of change questionnaire^(22)^, defined as being pre-contemplation), or inability to provide informed consent.

### Randomisation and blinding

Participants who were deemed eligible and gave consent to take part in the study were randomised to one of three intervention arms at a ratio of 1:1:1, using a randomisation scheme generated from www.randomization.com with a block size of nine. The three intervention groups were (i) a peer support intervention, (ii) a dietitian-led support intervention or (iii) a minimal support intervention. It was envisaged that recruitment for this trial would be phased, and the delivery of the peer and dietitian-led support interventions would proceed using a wait-list approach, when a minimum of six eligible participants were randomised to each of these intervention group arms. Participants were then invited for the baseline assessments. After baseline data were collected, participants were then informed of their allocated intervention group (concealed within an opaque envelope). Due to the nature of the study, it was not possible to blind study participants or members of the research team; however, laboratory and data analysis and assessment of primary outcome were carried out by an investigator blinded to treatment allocation.

### Trial to Encourage Adoption and Maintenance of a MEditerranean Diet study interventions

The three TEAM-MED interventions varied in the intensity and nature of support provided to encourage adoption of dietary behaviours consistent with an MD. The dietary behaviours included increased consumption of wholegrain cereal foods, fruit, vegetables, fish (particularly oily fish), legumes, unprocessed nuts, olive-oil and/or rapeseed oil and olive-oil based spreads; decreased consumption of red and processed meat and moderate alcohol consumption (if already being consumed). Rapeseed oil was permitted alongside olive-oil, considering the similar fatty acid composition, but reduced cost in Northern Ireland. Moderate alcohol consumption was permitted if participants already consumed alcohol.

All study participants were provided with written MD educational materials. These included an information booklet on following an MD (outlining what the MD is, health benefits, and general advice and tips for following), suggested meal plans, shopping lists and seasonal recipe books developed specifically for the study to support MD behaviour change. Details of the further support received in each of the three intervention groups are detailed below, and in the intervention development paper^(19)^ and protocol^(20)^.

#### The peer support group

A full description of the theory-based, tailored peer support intervention has been published elsewhere^(19)^. In the peer support group (PSG), participants were scheduled to attend a group programme consisting of eleven group sessions over the 12-month period, delivered by two trained peer supporters, whose training has been described elsewhere^(20)^. Groups involved up to ten study participants who met in a convenient location within the community setting. Each group session lasted up to 2 hours and included a brief (10–15 min) MD and/or behavioural education component delivered by peer supporters and designed to provide a focus for group discussion. The group topics included ‘health benefits of an MD’, ‘changing fat intake’, ‘eating more wholegrain’ and ‘eating a seasonal MD’. Practical food demonstrations (via food tasting sessions) were also included in four of the group sessions. Personal weigh-in and blood pressure measurements were available in each peer-support session, with feedback offered by a peer supporter.

In addition to the MD educational materials previously described, a personal workbook was given to participants at the beginning of the group programme to facilitate dietary goal-setting and self-monitoring of personal dietary goals^(24)^. Participants were also encouraged to maintain contact with other group members and the peer supporters in between sessions to promote social support and group cohesion.

#### The dietitian-led support group

The intervention in the dietitian-led support group (DSG) was based on that reported in the PREDIMED study, which has been shown to be effective in achieving increased adherence to MD^(8)^. Participants attended, at baseline, an individual face-to-face 90 min motivational interview with a study dietitian, where personal MD dietary goals were agreed and participants having received the educational materials previously described.

In addition, participants were provided with key MD foods over the 12-month intervention for daily consumption based on their personal preference for either:

- 50 ml extra virgin olive oil or,
- 30 g nuts (15 g walnuts, 7·5 g almonds and 7·5 g hazelnuts) or,
- a combination of both (25 ml extra virgin olive oil plus 15 g nuts (8 g walnuts, 3·5 g almonds and 3·5 g hazelnuts).

Participants were scheduled to attend a 2-h quarterly structured group education session (at 3, 6, 9 and 12 months) led by the dietitian, with up to four other study participants. The four-group session topics were ‘changing to an MD’, ‘enjoying fruit and vegetables’, ‘eating more wholegrain’ and ‘continuing to eat an MD’. After each group session, the dietitian provided a 15 min individual progress review and feedback. Throughout the intervention period, and between group sessions, participants had unlimited telephone/email contact with the dietitian for on-going support to optimise compliance with the intensive support MD intervention.

#### The minimal support group

Participants allocated to the minimal support group (MSG) received, at baseline, the same TEAM-MED study written MD educational materials as the other two groups. Participants were offered an individual appointment with a researcher for personal MD advice at the end of the intervention period. This group served as a control group.

### Outcomes assessed

The TEAM-MED study’s primary outcome was dietary behaviour change, with the target behaviour change being defined as ≥ 3-point increase in MDS from baseline to 6 months. Other measured outcomes included nutritional biomarkers as indicators of MD adherence, health markers, including CVD risk factors (e.g. body weight, blood pressure) and markers of diabetes risk (blood glucose and glycated haemoglobin (HbA1c) levels). Study recruitment strategies, attrition and retention rates were collected, as well as data collected to inform the estimation of the sample size required for a large-scale trial (MDS standard deviation and retention rates). A process evaluation, including exploration of possible mediators of MD behaviour change in response to the intervention and contextual factors that could influence implementation of the intervention or moderate the study outcomes, will be published separately.

### Study assessments

Study assessments were conducted face-to-face by a trained study researcher, at baseline, 3, 6 and 12 months. Each assessment visit followed strict standardised operating procedures, lasted approximately 3·5 h, and took place in dedicated research facilities in Belfast. Each participant attended the same facility for all four appointments. The assessments detailed below were carried out at each of the four study visits, in the same manner for all participants (regardless of intervention arm).

#### Mediterranean diet behaviour change

A fourteen-item MDS questionnaire was used to determine MD adherence and ease of adoption of dietary behaviours consistent with MD. This was based on a similar questionnaire used in the PREDIMED study^(8,21)^ and adapted to accommodate Northern European population food choices. The TEAM-MED pilot study utilised data from Logan *et al.* ^(13)^ to validate a locally adapted MDS with MDSs from previous studies^(2,25–27)^. The validation analysis demonstrated moderate agreement between the MDS used in PREDIMED and the TEAM-MED MDS. It also showed a significant association between the TEAM-MED MDS and other MDSs and nutritional biomarkers indicating that it provides a potentially valid tool for collecting data on MD adherence in a Northern European population^(28)^.

#### Anthropometric assessments

Standing height was measured to the nearest 0·1 cm using a wall-mounted stadiometer and body weight with a calibrated digital weighing scale (Tanita HS-301, Tanita, Yiewsley, UK) to the nearest 0·1 kg, and these were used to calculate BMI (kg/m^2^).

#### Clinical and biochemical measures

Blood pressure (mmHg) was measured in the dominant arm, after a 5-minute rest in a seated position, using a calibrated automated sphygmomanometer (Omron M5–1, UK). Three separate blood pressure measurements were recorded over a 5-minute period, and mean systolic and diastolic blood pressure were calculated from the second and third readings.

Blood samples were collected after an overnight fast and separated and stored in aliquots at −80°C until analysis. Fasting plasma glucose and 2-h plasma glucose (after a 75 g oral glucose load) were measured using an automated glucose oxidase method on a Beckman Glucose Analyzer 2, while HbA1c and a fasting lipid profile were measured using commercially available kits on an ILab-600 biochemical analyser (Instrumentation Laboratory). In addition, a panel of nutrient biomarkers was measured in plasma/serum samples to reflect the MDS food group targets^(13)^ and overall compliance with the MD. Serum concentrations of carotenoids were measured by reverse phase HPLC as described by Craft^(29)^. Plasma vitamin C was measured on a FLUOstar Optima plate reader (BMG Labtech) adapted from the method by Vuilleumier & Keck^(30)^. Finally, plasma fatty acids including EPA were assessed by GC^(31,32)^. The intra-assay CV for each of the nutrient biomarkers was < 10 %.

### Sample size and statistical analyses of pilot study

In pilot studies, a formal sample size calculation is not required; however, there is some debate regarding the sample required for pilot studies^(33)^, and we were able to estimate likely power based on data on the variability of MDS results (sd 1·3 from baseline to 6 months) in a behavioural counselling group of a previous trial in patients with existing CVD^(13)^. From this, a study of twenty-five subjects per group would have 90 % power to detect a difference between treatment groups in mean MDS change from baseline to 6 months (adoption phase) of 1·4 units and a difference in mean score change from 6 months to 12 months (maintenance phase) of 1·0 units. A 30 % drop-out rate during the study period was also assumed. Given that TEAM-MED was evaluating a different intervention to encourage MD adherence in a different population, the data generated in this pilot study should provide a more reliable sample size calculation for a definitive study.

Analyses relating to MDS have been conducted using *original* MDS data (a complete case analysis), comprising all patients who were randomised to one of the intervention arms and who had all post-randomisation endpoint data recorded. Analyses relating to MDS were also conducted using *augmented* MDS data. This was carried out to account for withdrawals; missing values were imputed for withdrawals using measurements at a prior time point. Regression analysis using the immediately preceding MDS score and treatment group, as well as the interaction between these two variables, was performed and predictions from the regression provided the imputed values.

Hypothesis testing is not usually conducted in pilot studies so what was conducted was exploratory to see if the intervention shows promise in terms of changing MDS and whether this differed over time by intervention group, although with the understanding that the power to conduct these analyses was uncertain, and that the main objectives were to estimate variability and formally conduct sample size^(34)^. Repeated measures ANOVA was conducted on both the original and augmented MDS data to examine the indicative effect of the TEAM-MED intervention on change in MDS. This was conducted to assess the effect of the intervention over time and to investigate the interaction between treatment group and time to ascertain any indicative differences in MD adherence between study groups. As MDS was the study’s primary outcome, an ANCOVA was conducted to further investigate the differences in MDS between the PSG and either DSG or MSG at each time-point and with either screening or baseline MDS used as a covariate.

Repeated measures ANOVA were also conducted to examine changes in nutritional biomarkers as indicators of MD adherence and biomarkers of CVD and T2DM risk to investigate any effects of the TEAM-MED intervention on these factors. Some of the biomarker measurements required log transformation, and these results are presented as geometric means and 95 % CI. All repeated measures ANOVA tests are presented with a Greenhouse–Geisser correction for lack of sphericity^(35)^.

Pearson correlation coefficients were calculated to investigate if there were any associations between change in augmented MDS between baseline and 6 months and baseline and 12 months, and change in nutritional biomarkers, CVD and T2DM risk factors, between baseline and 6 months and baseline and 12 months, i.e. to determine if increased adherence was associated with increased biomarker and CVD and T2DM risk factor response.

Statistical analyses were conducted using SPSS version 22 with statistical significance set at *P* ≤ 0·05.

## Results

### Recruitment and retention

**Fig. 1.**
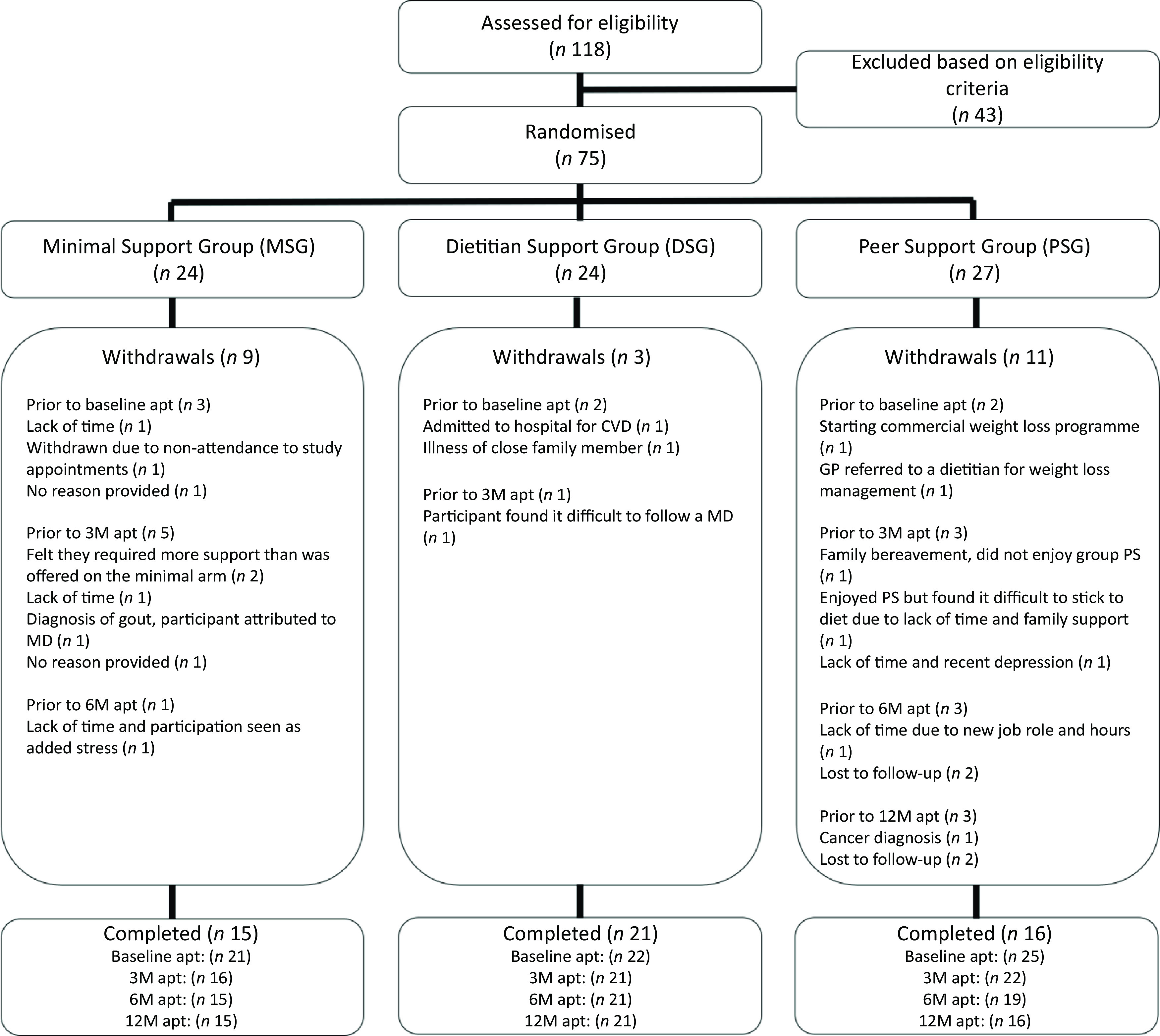
CONSORT diagram of the flow of participants through the The Trial to Encourage Adoption and Maintenance of a MEditerranean Diet (TEAM-MED) study.

Recruitment for the TEAM-MED study began in October 2014 and was completed in April 2015, and, as the study had a 12-month duration, the last study appointment took place in April 2016. The Consort diagram in Fig. 1 demonstrates the flow of participants through the TEAM-MED trial. Totally, 118 individuals were assessed for eligibility at a screening appointment, with 43 (36 %) excluded from taking part due to pre-defined criteria, leaving 75 (64 %) potential participants eligible for randomisation to one of three intervention arms. From the eligible screened volunteers, there were 7 (9 %) withdrawals prior to baseline data collection.

After 3 months, there were retention rates of 67 %, 88 % and 81 % in the MSG, DSG and PSG, respectively. This led to an overall retention rate of 79 % at 3 months. After 6 months, there were retention rates of 63 %, 88 % and 70 % in the MSG, DSG and PSG, respectively. This led to an overall retention rate of 73 % at 6 months. At the end of the 12-month period, there were retention rates of 63 %, 88 % and 59 % in the MSG, DSG and PSG, respectively, who completed the trial, representing an overall retention rate of 69 % at 12 months. At the end of the 12-month period, there had been twenty-three withdrawals from the study, and reasons for this can be seen in Fig. 1. Overall, the rate of withdrawals, at 31 %, was slightly higher than the rate of 30 % assumed during study planning.

### Descriptive characteristics of study participants

Baseline characteristics of the study participants as a whole and according to intervention group are presented in Table 1. Of the seventy-five individuals who were recruited into the study, 56 % (*n* 42) were male, with a mean age of 57·1 years. About 78·7 % of these seventy-five individuals were classed as obese, with a mean BMI of 33·5 kg/m^2^ for the whole group. The average time spent in full time education was 13·5 years. Participants in the PSG had a higher mean BMI compared with the other two groups (Table 1). All other baseline characteristics were similar between the three intervention groups.

**Table 1.** Descriptive characteristics of participants in the three intervention groups as part of the The Trial to Encourage Adoption and Maintenance of a MEditerranean Diet (TEAM-MED) study (Mean values and standard deviations; numbers and percentages)

	Whole study population (*N*_max_ = 75)		Minimal Support Group (*N*_max_ = 24)		Dietitian Support Group (*N*_max_ = 24)		Peer Support Group (*N*_max_ = 27)	
	Mean	sd	Mean	sd	Mean	sd	Mean	sd
Gender								
Male								
*n*	42		11		16		15	
%	56		46		67		56	
Age (at baseline)	57·1	6·7	58·0	5·1	57·8	8·1	55·7	6·6
Years spent in full time education	13·5	2·9	13·5	3·0	13·6	3·4	13·4	2·5
Marital status (at baseline)								
Married/co-habiting								
*n*	43		12		15		16	
%	57		50		63		59	
BMI (at screening) (kg/m^2^)	33·5	4·4	32·8	4·0	31·9	3·8	35·6	4·5
MDS (at screening)	2·1	0·9	2·0	0·9	2·4	0·7	1·9	1·0

MDS-Mediterranean Diet Score

### Effect of interventions to encourage Mediterranean diet adoption on the Mediterranean Diet Score

At screening (Table 1), there were no significant differences between intervention groups in MDS. Analyses of the MDS data from all time points after screening were performed using both *original* data and *augmented* data (with imputation for missing values).

Table 2 shows results from a repeated measures ANOVA to investigate whether there were differences in MDS over time and between the intervention groups over time. For both the original and augmented analyses, there was a significant effect of time on MDS when considering all of the intervention groups (*P* < 0·001), with MDS increasing in all groups over the 12 months. The target behaviour change for the intervention was defined as a ≥ 3 point increase in MDS from baseline to 6 months, with this being maintained at 12 months. From baseline to 6 months, MDS (using the original values) increased from 2·67 to 6·92 in the MSG, from 3·32 to 8·95 in the DSG and from 4·12 to 8·84 in the PSG, and these increases were largely maintained at 12 months indicating that, in general, the target behaviour change was attained in all intervention groups. The interaction test showed no significant difference between the groups over time in the original analysis; however, a significant difference over time between groups (*P* = 0·003) was apparent in analysis with the augmented MDS and a reduction in MDS by 12 months was suggestive of poorer maintenance in the PSG group.

**Table 2.** Differences in complete and augmented Mediterranean diet score between the intervention groups and over the 12 month intervention period (Mean values and 95 % confidence intervals)

		MSG (*n* 11)		DSG (*n* 16)		PSG (*n* 20)		Greenhouse-Geisser corrected *P* value	
		Means	95 % CI	Means	95 % CI	Means	95 % CI	Effect of time	Treatment group by time interaction
MDS original	Baseline	2·67	2·05, 3·28	3·32	2·59, 4·05	4·12	3·10, 5·14	< 0·001	0·13
	3 months	7·29	6·29, 8·28	8·50	7·70, 9·30	8·43	7·22, 9·64		
	6 months	6·92	5·70, 8·14	8·95	8·11, 9·80	8·84	7·57, 10·11		
	12 months	7·33	5·95, 8·72	8·90	8·04, 9·77	8·13	6·68, 9·57		
		MSG (*n* 24)		DSG (*n* 24)		PSG (*n* 27)			
MDS augmented	Baseline	2·71	2·16, 3·26	3·38	2·71, 4·04	4·07	3·13, 5·02	< 0·001	0·003
	3 months	7·50	6·90, 8·10	8·54	7·88, 9·20	8·52	7·56, 9·48		
	6 months	6·96	6·33, 7·59	8·96	8·23, 9·69	8·37	7·32, 9·42		
	12 months	7·21	6·37, 8·04	8·92	8·17, 9·66	7·41	6·38, 8·43		

MDS, Mediterranean Diet Score; PSG; peer support group, DSG, dietitian support group, MSG, minimal support group. Results obtained from repeated measures ANOVA and presented as means (95 % CI). Augmented refers to data that has had missing values imputed.

ANCOVA was used to further investigate whether there were any differences in MDS between the intervention groups at specific time points using both original (Table 3) and augmented (Table 4) values. At baseline, in the analysis using original and augmented values, there were significant differences in MDS, with those in the PSG having significantly higher MDS than the MSG (*P* = 0·01 and *P* = 0·002 respectively) and DSG (*P* = 0·02; *P* = 0·01; original and augmented data, respectively), which was likely due to delays caused by the wait-list approach used during the formation of the peer support groups (where groups were not formed until at least six participants in the same geographical area had been randomised to the peer support group to allow their travel to these groups to be achievable). Therefore, based on this observation, two ANCOVAs were performed, one adjusted for screening MDS values and one adjusted for baseline MDS values for completeness.

**Table 3.** Comparisons of *original* Mediterranean diet score between the intervention groups at specific time points (Mean values and 95 % confidence intervals)

Timepoint	Covariate	*n*			Intervention group comparisons					
					PSG *v.* MSG			PSG *v.* DSG		
		MSG	DSG	PSG	Mean difference	95 % CI	*P* value	Mean difference	95 % CI	*P* value
Baseline	Screening	21	22	25	1·49	0·48, 2·51	0·01	1·20	0·18, 2·20	0·02
3 months	Screening	14	20	21	0·99	–0·38, 2·37	0·15	0·16	–1·09, 1·41	0·80
6 months	Screening	13	21	19	1·92	0·37, 3·47	0·02	0·14	–1·25, 1·52	0·85
12 months	Screening	15	21	16	0·63	1·00, 2·27	0·44	–0·67	–2·17, 0·84	0·38
3 months	Baseline	14	20	21	0·37	–1·05, 1·79	0·60	–0·30	–1·52, 0·92	0·63
6 months	Baseline	13	21	19	1·41	–0·16, 2·97	0·08	–0·33	–1·67, 1·01	0·63
12 months	Baseline	15	21	16	–0·08	–1·75, 1·59	0·92	–1·15	–2·62, 0·32	0·12

PSG, peer support group; DSG, dietitian support group; MSG, minimal support group. Results obtained from ANCOVA, with either screening or baseline MDS included as a covariate and presented as mean differences (95 % CI).

**Table 4.** Comparisons of augmented Mediterranean diet score between the intervention groups at specific time points (where missing values have been imputed)

Timepoint	Covariate	*n*			Intervention group comparisons					
					PSG *v.* MSG			PSG *v.* DSG		
		MSG	DSG	PSG	Mean difference	95 % CI	*P* valu*e*	Mean difference	95 % CI	*P* value
Baseline	Screening	24	24	27	1·44	0·53, 2·35	0·002	1·19	0·25, 2·13	0·01
3 months	Screening	24	24	27	1·07	0·08, 2·07	0·03	0·34	–0·68, 1·36	0·50
6 months	Screening	24	24	27	1·46	0·35, 2·57	0·01	–0·28	–1·42, 0·86	0·63
12 months	Screening	24	24	27	0·26	–0·89, 1·41	0·66	–1·12	–2·30, 0·07	0·06
3 months	Baseline	24	24	27	0·34	–0·63, 1·31	0·49	–0·37	–1·31, 0·57	0·43
6 months	Baseline	24	24	27	0·87	–0·26, 2·00	0·13	–0·86	–1·96, 0·23	0·12
12 months	Baseline	24	24	27	–0·47	–1·63, 0·69	0·42	–1·85	–2·97, −0·73	0·002

PSG, peer support group, DSG, dietitian support group, MSG, minimal support group. Results obtained from ANCOVA, with either screening or baseline MDS included as a covariate and presented as mean differences (95 % CI).

In the analysis using original MDS values (Table 3), there were significant differences in MDS between the PSG and MSG at 6 months, with the PSG having a significantly higher MDS at this time point (*P* = 0·02), adjusting for screening, although this did not reach significance when adjusting for baseline MDS (*P* = 0·08). However, by 12 months, there were no statistically significant differences in MDS between PSG and MSG. There were no significant differences between PSG and DSG at any timepoint.

In the augmented analysis (Table 4), significant differences were observed between the PSG and MSG at 3 and 6 months, with the PSG having significantly higher scores (*P* = 0·03 and *P* = 0·01, respectively), when adjusting for screening MDS, but not when adjusting for baseline. There were no significant differences in MDS between PSG and MSG at 12 months. The PSG had a significantly lower MDS compared with the DSG at 12 months (*P* = 0·002 when adjusting for baseline MDS; *P* = 0·06 when adjusting for screening MDS).

In summary, both the original and augmented analyses suggest that all intervention groups increased adherence towards an MD over the 12-month intervention. Although analyses are only indicative, the PSG and DSG produced larger changes in MDS than in the MSG at 6 months, with some suggestion that the DSG supported maintenance of behaviour change more than the PSG at 12 months.

### Increased Mediterranean diet adherence and nutritional biomarkers

Table 5 shows the results from repeated measures ANOVA investigating differences in selected MD-associated nutritional biomarkers, both over the course of the intervention and between the intervention groups over the study period. Across the whole study population, there was a statistically significant difference over time in plasma vitamin C concentrations (*P* = 0·01), and this approached significance for *β*-cryptoxanthin and EPA. However, there was no statistically significant difference between the intervention groups over time. There were no other statistically significant differences over time or between the intervention groups over time for the other biomarkers investigated.

**Table 5.** Differences in nutritional biomarkers and fatty acids between the intervention groups and over the 12-month intervention period

		MSG		DSG		PSG		Greenhouse-Geisser corrected *P* value	
		Mean	95 % CI	Mean	95 % CI	Mean	95 % CI	Effect of time	Treatment group by time
Vitamin C (µmol/l)	*n*	11		14		11		0·01	0·60
	Baseline	25·1	18·2, 32·1	25·2	19·4, 31·0	24·8	19·1, 30·5		
	3 months	26·4	21·5, 31·4	29·9	24·9, 34·9	30·1	24·3, 35·8		
	6 months	20·9	17·2, 24·5	28·2	22·6, 33·8	24·5	19·6, 30·3		
	12 months	22·9	18·3, 27·5	26·7	21·4, 32·0	27·1	19·7, 34·4		
Lutein (µmol/l)*	*n*	12		16		13		0·16	0·75
	Baseline	0·18	0·13, 0·22	0·23	0·18, 0·27	0·17	0·12, 0·21		
	3 months	0·17	0·13, 0·21	0·23	0·19, 0·27	0·20	0·13, 0·28		
	6 months	0·18	0·14, 0·22	0·23	0·18, 0·27	0·25	0·15, 0·34		
	12 months	0·17	0·14, 0·21	0·22	0·18, 0·26	0·20	0·13, 0·27		
*β*-cryptoxanthin (µmol/l)*	*n*	12		16		13		0·07	0·67
	Baseline	0·08	0·05, 0·10	0·10	0·07, 0·13	0·07	0·05, 0·10		
	3 months	0·07	0·06, 0·08	0·11	0·07, 0·15	0·08	0·05, 0·12		
	6 months	0·08	0·06, 0·10	0·13	0·08, 0·17	0·08	0·04, 0·12		
	12 months	0·07	0·05, 0·08	0·11	0·08, 0·14	0·09	0·05, 0·13		
EPA (% of total)*	*n*	10		16		12		0·08	0·59
	Baseline	1·43	0·82, 2·05	1·26	0·75, 1·77	1·15	0·86, 1·44		
	3 months	1·41	0·91, 1·91	1·40	1·14, 1·66	1·55	1·25, 1·85		
	6 months	1·61	1·01, 2·22	1·58	1·18, 1·98	1·43	1·10, 1·76		
	12 months	1·10	0·86, 1·33	1·46	1·11, 1·82	1·35	1·00, 1·70		

PSG, peer support group; DSG, dietitian support group; MSG, minimal support group.

* Variable required log transformation and presented as geometric mean (95 % CI). Results obtained from repeated measures ANOVA and presented as means (95 % CI).

### Increased Mediterranean diet adherence and CVD and type 2 diabetes mellitus risk factors

Table 6 shows the results from repeated measures ANOVA investigating differences in CVD and T2DM risk factors, both over the course of the intervention and between the intervention groups over the study period. Across the study population as a whole, BMI, HbA1c levels, systolic and diastolic blood pressure were found to be significantly different over time (*P* = 0·01, *P* < 0·001, *P* = 0·02 and *P* = 0·01, respectively). However, there was no statistically significant difference in change in these risk factors between the intervention groups over the study period. No other differences were observed over time or between the intervention groups over time for the other measures investigated (fasting glucose and post OGTT, lipids).

**Table 6.** Differences in CVD and type 2 diabetes mellitus (T2DM) risk factors between the intervention groups and over the 12-month intervention period (Mean values and 95 % confidence intervals)

		MSG		DSG		PSG		Greenhouse-Geisser corrected *P* value	
		Mean	95 % CI	Mean	95 % CI	Mean	95 % CI	Effect of time	Treatment group by time
HDL (mmol/l)	*n*	10		15		12		0·74	0·34
	Baseline	1·40	1·25, 1·55	1·42	1·28, 1·56	1·46	1·29, 1·62		
	3 months	1·36	1·22, 1·51	1·47	1·32,1·62	1·43	1·26, 1·61		
	6 months	1·31	1·09, 1·53	1·53	1·32, 1·75	1·43	1·22, 1·64		
	12 months	1·34	1·23, 1·48	1·50	1·35, 1·66	1·48	1·30, 1·66		
TAG (mmol/l)*	*n*	10		15		12		0·20	0·41
	Baseline	2·36	1·66, 3·06	1·75	1·50, 1·99	1·73	1·23, 2·23		
	3 months	2·23	1·57, 2·88	1·66	1·31, 2·01	1·82	1·25, 2·38		
	6 months	2·31	1·57, 3·06	1·57	1·25, 1·89	1·73	1·37, 2·09		
	12 months	2·47	1·83, 3·10	1·68	1·33, 2·03	1·59	1·25, 1·94		
Cholesterol (mmol/l)	*n*	10		15		12		0·15	0·22
	Baseline	6·03	5·25, 6·82	5·83	5·22, 6·43	6·03	5·51, 6·56		
	3 months	5·67	4·88, 6·46	5·68	5·01,6·36	5·84	5·11, 6·58		
	6 months	5·53	4·54, 6·52	5·78	5·14, 6·43	5·83	5·28, 6·38		
	12 months	5·95	5·18, 6·72	5·70	5·07, 6·32	5·65	5·08, 6·21		
HbA1c (%)	*n*	9		13		10		< 0·001	0·70
	Baseline	5·67	5·45, 5·88	5·56	5·36, 5·76	5·65	5·48, 5·82		
	3 months	5·63	5·39, 5·88	5·46	5·30,5·63	5·38	5·03, 5·74		
	6 months	5·65	5·38, 5·91	5·46	5·32, 5·60	5·56	5·40, 5·72		
	12 months	5·64	5·44, 5·85	5·45	5·31, 5·60	5·56	5·42, 5·69		
Fasting blood glucose (mmol/l)	*n*	10		15		11		0·28	0·80
	Baseline	5·81	5·32, 6·31	5·76	5·37, 6·15	5·88	5·49, 6·26		
	3 months	6·22	5·54, 6·90	5·57	5·34, 5·80	5·98	5·42, 6·55		
	6 months	6·04	5·46, 6·62	5·67	5·46, 5·89	5·70	5·45, 5·95		
	12 months	5·82	5·38, 6·27	5·545	5·31, 5·78	5·80	5·50, 6·09		
Blood glucose post oral glucose tolerance test (mmol/l)	*n*	8		11		7		0·93	0·35
	Baseline	6·95	5·72, 8·17	7·05	5·78, 8·32	7·54	6·48, 8·60		
	3 months	8·26	6·72, 9·79	6·52	5·32, 7·72	8·36	6·60,10·12		
	6 months	7·61	5·83, 9·39	6·53	4·93, 8·12	7·00	5·54, 8·46		
	12 months	7·73	6·24, 9·22	6·99	5·77, 8·22	7·55	6·36, 8·74		
BMI (kg/m^2^)	*n*	11		20		16		0·01	0·71
	Baseline	32·36	30·86, 33·86	31·88	30·05, 33·71	35·41	33·46, 37·35		
	3 months	33·00	30·92, 35·09	31·25	29·42, 33·07	34·87	32·63, 37·11		
	6 months	32·55	30·87, 34·24	31·19	29·42, 32·97	34·49	32·14, 36·84		
	12 months	32·93	31·12, 34·75	31·42	29·68, 33·16	35·32	32·75, 37·88		
Systolic blood pressure (mmHg)	*n*	11		20		16		0·02	0·67
	Baseline	141·00	133·68, 148·32	135·36	128·15, 142·58	138·66	131·87, 145·45		
	3 months	143·68	137·84, 149·51	127·48	121·75, 133·20	136·19	129·16, 143·22		
	6 months	144·00	132·98, 155·02	132·69	125·44, 139·94	136·92	128·63, 145·22		
	12 months	137·26	130·35, 144·17	129·98	123·94, 136·01	136·16	127·13, 145·19		
Diastolic blood pressure (mmHg)	*n*	11		20		16		0·01	0·43
	Baseline	89·52	84·29, 94·76	87·43	82·99, 91·88	89·30	83·44, 95·16		
	3 months	91·54	85·39, 97·68	81·38	77·06, 85·69	86·67	81·64, 91·70		
	6 months	88·27	80·24,96·30	82·95	78·06, 87·84	82·71	77·64, 87·78		
	12 months	86·30	80·99,91·61	81·83	77·40, 86·27	87·47	81·81, 93·13		

PSG, peer support group; DSG, dietitian support group; MSG, minimal support group.

* Variable required log transformation and presented as geometric mean (95 % CI). Results obtained from repeated measures ANOVA and presented as means (95 % CI).

### Associations between change in Mediterranean Diet Score and change in nutritional biomarkers and CVD and type 2 diabetes mellitus risk factors

Negative associations were observed between change in MDS and change in BMI between baseline and 12 months (*r* = –0·33; *P* = 0·017). A negative association was also observed between change in MDS between baseline and 12 months and change in levels of HbA1c (*r* = –0·39; *P* = 0·014), and this approached statistical significance for SBP (*r* = –0·25, *P* = 0·07). Positive associations were observed between change in MDS between baseline and 12 months and change in levels of Vitamin C (*r* = 0·30; *P* = 0·049) and this approached significance for serum lutein (*r* = 0·26; *P* = 0·07). Similar patterns were observed for the change in outcomes *v* change in MDS at 6 month data (data not shown). The focus was on 12-month data as that allowed for maximum time for dietary change to impact on the outcomes assessed.

### Sample size for a future trial

Pilot study results were used to estimate the size for a future two-group equivalence trial of PSG and DSG in a parallel group design. MDS at 12 months was taken as the primary outcome with baseline MDS used as a covariate. PASS2008^(36)^ gave a sample size of *n* 111 per group to achieve 90 % power at a 5 % significance level, with equivalence limits set at −1·00 and 1·00, when the true difference between the means was assumed to be 0·00 and the standard deviation was 2·25, as observed in the analysis of covariance of the current pilot trial. This was then increased to account for an anticipated dropout rate of 28 %, as recorded in these two groups in the pilot study, resulting in a final trial size estimate of 154 per group or 308 in total.

## Discussion

The TEAM-MED pilot trial aimed to explore to explore methods of increasing MD adoption in a non-Mediterranean population at high risk of CVD.

### Feasibility considerations

One of the main aims of the study was to explore feasibility, specifically testing recruitment strategies and retention and attrition rates. During the course of the trial, two main changes were made to the protocol designed to influence and improve recruitment. Recruitment methods were extended from advertisements on radio stations, in church bulletins, on health service intranets and other platforms to also directly contacting potentially eligible participants via letter via GP surgeries. Changes were also made to the CVD risk criteria, calculated using JBS CVD risk prediction charts and where certain clinical data were unavailable, and this is detailed within methods.

Other than the changes described above, recruitment methods were as originally intended and recruitment targets were met within the intended timescale. However, of those individuals who were excluded from the intervention at the screening appointment (*n* 43 out of a total of 118 screened), 65 % were due to either having an MDS of ≥ 3, a CVD risk of below 20 % over 10 years or a combination of these. This is a particularly high rate of exclusion and would suggest that the inclusion and exclusion criteria might have to be reassessed before a future definitive trial.

The current study also allowed recruitment (64 %), retention (73 % at 6 months and 69 % at 12 months) and withdrawal (31 %) rates to be calculated. Totally, seventy-five participants (twenty-five participants per intervention group) were recruited to the trial and randomised to one of the intervention arms, and the study had a completion rate of 69 %. The withdrawal rate of 31 % was slightly higher but similar to the initial 30 % assumed for the power calculation, and these figures can be used to inform the definitive trial sample size, and withdrawal rates did differ by group, being lower in the DSG compared with the MSG and PSG. Why withdrawal rates were higher in the PSG than in the DSG (59 % *v* 88 % at 12 months, respectively) is uncertain, but may be due to, for example, the contact with a health professional or supplementary food provision, and this will be explored further within the accompanying process evaluation.

An augmented analysis was conducted to account for the withdrawals and to observe the effect withdrawals may have had on the MDS variability results. However, in a definitive future trial, study protocol adaptations to limit withdrawals, such as considering an incentive for completion should be considered, and, again, the process evaluation will inform this.

### Mediterranean Diet Score variability data

There are a number of already demonstrated benefits of adhering to MD, including reducing the risk of developing CVD and other non-communicable diseases such as T2DM^(2,37,38)^. In this study, all intervention groups, in both the augmented and original analyses, increased their MDS over the intervention period. Median increases of between 3 and 5 points were observed between baseline and 12 months for all of the intervention groups in the current study, suggesting a marked increase in adherence, accompanied by some shifts in objective biomarkers related to an MDS, and also CVD and T2DM risk factors (BMI, systolic and diastolic blood pressure and HbA1c), highlighting the potential benefits of increasing MD adherence on health outcomes in this population. Furthermore, statistically significant associations were observed between increasing MDS over the intervention period and decreasing BMI and HbA1c, suggesting that more marked dietary behaviour change was associated with greater improvement in these outcomes, Both these analyses suggest a benefit of consuming an MD on CVD and T2DM health outcomes, and similar findings have been shown previously^(39–41)^. Increases of two points in MDS have been associated with a 10 % reduction in CVD risk^(2)^, and therefore the observed increases are likely to be of clinical benefit. Whilst no changes in fasting glucose and lipid profiles were observed, this might not be surprising given that baseline levels were not significantly elevated. The inclusion of rapeseed oil in the guidance as an alternative to olive oil may also have reduced any likely impact on the lipid profile outcomes, and this will be explored in the process evaluation.

There is less available evidence supporting a CVD benefit of MD adherence in non-Mediterranean countries compared with Mediterranean countries^(42)^, and so the current observation suggests that the potential health benefits (assessed by measurement of CVD risk factors) may be similar, even at lower initial levels of intake (MDS score in the current study was 2 on a fourteen point scale, compared with > 8 in PREDIMED on a similar scale)^(8)^. One exception is the MedLey study in older Australians that demonstrated improved CVD risk factors (blood pressure, TAG and F2-isoprostanes) as well as greater flow-mediated dilatation^(43,44)^ after a 6-month MD intervention. Dietary behaviour change was achieved in that study using more intensive methods than used with our PSG and more similar to PREDIMED and our DSG: dietetic consultations and motivational interviewing at 3 and 6 months and every fortnight in between, provision of written educational materials, sample menus and recipes, a daily food checklist to help track compliance with the diet and provision of key foods^(45)^.

As this was a pilot study and not powered to detect differences between intervention groups, it is unclear as to whether the PSG would be as effective at increasing and maintaining adherence to MD when compared with the more intensive DSG and less intensive MSG, although the data were explored for indicative effects. These results do, however, highlight that it is possible for adults at a high risk of developing CVD in a non-Mediterranean population to adopt and maintain an MD in response to an MD intervention and provides variability data which can be used to inform a future, definitive trial.

As it was only in the augmented analysis that a significant difference in MDS was observed between the intervention groups over time, this could suggest a potential impact of differing levels of withdrawal on the MDS results. However, it could also suggest a potential difference in the impact of the different interventions on MDS adoption, with more intensive interventions yielding the observed larger and maintained increases in MDS, which will lead to increased dietary compliance and associated potential long-term cardiovascular health benefits. The DSG, the most intensive intervention, was based on the dietitian led intervention used by Estruch *et al.* ^(8)^ in the PREDIMED intervention, and this has been shown previously to be effective at improving adherence to MD when compared with a control low-fat diet. The DSG included dietitian-led sessions and provision of key MD foods; therefore, this intervention is likely to be the most costly on an ongoing basis, and less likely to be applied at a population level. The theory-based, tailored peer support intervention was developed as a lower cost intervention that would be as effective at encouraging dietary behaviour change towards an MD as the DSG, and data from this feasibility study certainly suggest that it produced increased MD adherence, although there was the suggestion that this was less likely to be maintained at 12 months when compared with the more intensive DSG, and the drop-out rate was greater in the PSG than the MSG and DSG groups. Initial development of the PSG intervention was expensive, requiring considerable staff time and resources over a lengthy development period, but, once developed, the PSG requires only the cost of training the peer supporters and their travel costs, thus ongoing costs would be substantially reduced compared to those of the DSG, although formal cost-effectiveness analysis was not conducted as part of this feasibility study. Peer-support-based interventions have been shown previously to be a cost-effective way to promote behaviour change^(46)^ and can lead to improvements in diet quality^(15–17,47,48)^.

While these findings indicate that behaviour change towards an MD is achievable in a non-Mediterranean population, there is still much to learn regarding the most cost-effective approaches to support MD behaviour change^(49)^, and particularly interventions to support longer-term maintenance of newly adopted MD behaviours in non-Mediterranean countries. The TEAM-MED trial has suggested that a less intensive and potentially more cost-effective dietary delivery method, via peer support, is feasible in a Northern European population at high CVD risk. The peer support intervention has been developed using a theory-based approach and is tailored to the needs of the target group to promote dietary behaviour change. Conclusions cannot be drawn from this pilot study regarding any formal comparison between the PSG and the DSG in terms of efficacy, and the above discussion is only based on indicative findings from this pilot study, but the data can be used to power a definitive trial to address this question. Supporting adoption and maintenance of a healthy diet such as the MD, which has been shown to effectively reduce the risk of cardiovascular risk factors and CVD, should be a high public health priority for disease prevention.

### Strengths and limitations

The TEAM-MED study can, as a pilot study which explored feasibility, guide the design of a definitive trial. The 12-month duration of the intervention, developed using MRC guidance for developing and evaluating complex interventions^(50)^, allowed assessment of both adoption and maintenance of MD. The MDS used was developed to ensure that it would not be only suitable for a non-Mediterranean population but also representative of MD to ensure comparison with other studies^(28)^. Nutritional biomarkers were also analysed to provide an objective assessment of dietary change, and this analysis is rarely conducted in studies of encouraging dietary behaviour change.

This study was designed as an RCT, the gold standard; however, it did have a number of limitations. While this was designed as a pilot trial to explore feasibility, formal progression criteria were not defined. Although BMI did seem to differ between the three groups at baseline, with the PSG having a higher mean BMI compared with the other two intervention groups, analysis was conducted to investigate whether this influenced the study’s primary outcome, and it did not have an impact. All three of the intervention groups increased their MDS over the study period and made significant changes towards an MD, and this could indicate that the MSG did not truly represent a control group in the RCT design. The MSG did receive detailed written information at baseline regarding encouraging adherence to an MD and, given the fact that they were screened for risk of CVD and deemed to be eligible, i.e. at high risk and willing to make dietary changes (according to screening stage of change questionnaire), this may have helped to encourage dietary change, and/or impacted dietary reporting.

For all of the intervention groups, increases in MDS were seen between screening and baseline assessments, and this increase was particularly apparent in the PSG. Due to the logistics involved in setting up group meeting venues to suit participants in the PSG and forming peer support groups, there was a particularly large time delay in beginning the intervention for participants in this group. Given that, at the screening appointment, the MD was explained to participants, the individuals in the PSG had more time than the other intervention groups to make changes towards their diet between screening and baseline measurements. Such an increase in the PSG meant that, although MDS was balanced across the intervention groups at screening, there were substantial differences between intervention groups at baseline assessment and this must be considered a study limitation.

MDS was assessed using a fourteen-item self-report questionnaire and is therefore subject to certain biases and misreporting, although researchers administered this questionnaire verbally, probing dietary habits in an attempt to record MDS as accurately as possible, and the reported changes in dietary behaviour were supported by the changes in objective measures of nutritional status related to MDS. Nutritional biomarkers specific for the overall MD pattern do not really exist, however, so what was measured represented some of the MD food groups (fruit and vegetables and fish). Food diaries were also completed by study participants and further analyses will explore whether reported MDS changes were consistent when measured by this alternative dietary assessment method, as well as exploring the pattern of food group changes associated with an increase in MDS. These analyses will also inform any specific changes to the dietary advice given, e.g. consuming vegetables with oil to maximised carotenoid absorption, or the use of olive oil over rapeseed oil.

Clearly, there are a number of behaviours involved in adopting an MD, and the process evaluation will include exploring ease of adoption across the range of targeted eating behaviours (foods and food groups) and the acceptability of the different elements of the peer support intervention. This approach will allow the intervention to be further adapted and tailored to the needs of the target population. A potential limitation of the peer support intervention is that it is designed to target only dietary behaviour change and does not consider broader lifestyle behaviours besides food consumption, such as physical activity and social interactions that are considered part of a Mediterranean lifestyle^(1)^ and are also important modifiable behaviours for CVD prevention^(51)^. Inclusion of these broader lifestyle behaviours will be considered within the study design for a definitive trial.

### Conclusion

The findings from this pilot trial indicate that a non-Mediterranean population at high CVD risk can adopt and maintain an MD and that a peer-support intervention is a feasible method to encourage this behaviour change. In this population, increasing adherence to an MD may also be associated with improved cardiovascular risk factor status. The variability data and insights from this pilot study will inform the design of a larger scale RCT where the efficacy and cost-effectiveness of the peer support intervention will be tested.
